# The Prevalence and Risks of Inappropriate Combination of Aspirin and Warfarin in Clinical Practice: Results From WARFARIN-TR Study

**DOI:** 10.4274/balkanmedj.2017.1472

**Published:** 2019-01-01

**Authors:** Salih Kılıç, Ahmet Çelik, Elif Çekirdekçi, Servet Altay, Deniz Elçik, Mehmet Kadri Akboğa, Mine Durukan, Çağrı Yayla, Mehdi Zoghi

**Affiliations:** 1 Department of Cardiology, Ege University School of Medicine, İzmir, Turkey; 2 Department of Cardiology, Mersin University School of Medicine, Mersin, Turkey; 3 Clinic of Cardiology, Tekirdağ Çorlu District State Hospital, Tekirdağ, Turkey; 4 Clinic of Cardiology, Edirne Sultan Murat 1. State Hospital, Edirne, Turkey; 5 Clinic of Cardiology, Ankara Research and Training Hospital, Ankara, Turkey; 6 Clinic of Cardiology, Türkiye Yüksek İhtisas Hospital, Ankara, Turkey; 7 Clinic of Cardiology, Mersin City Research and Training Hospital, Mersin, Turkey

**Keywords:** Anticoagulants, antiplatelet drugs, aspirin, inappropriate prescribings, Warfarin

## Abstract

**Background:**

The use of warfarin and aspirin in combination is restricted to limited patients under relevant guidelines.

**Aims:**

To evaluate the prevalence of the inappropriate combination of aspirin and warfarin therapy in daily practice and its risks.

**Study Design:**

Cross-sectional study.

**Methods:**

The awareness, efficacy, safety, and time in the therapeutic range of warfarin in the Turkish population study is a multi-center observational study that includes 4987 patients using warfarin for any reason between January 1, 2014, and December 31, 2014. To determine the prevalence of inappropriate combination use in daily practice, all patients who had a history of atherosclerotic disease (ischemic heart disease, peripheral artery disease) or cerebrovascular disease (n=1498) were excluded. The data of 3489 patients were analyzed. We defined inappropriate combination as all patients who received aspirin and warfarin regardless of the indication for warfarin use, under the direction of the European Society of Cardiology guideline recommendation.

**Results:**

The mean age of patients was 59.2±13.8 years (41.8% male). The prevalence of the inappropriate use of warfarin and aspirin combination was 20.0%. The prevalence of combination therapy in patients with a primary indication for mechanical heart valve, non-valvular atrial fibrillation, and other reasons was 20.5%, 18.7%, and 21.0%, respectively. Multivariate logistic regression analysis revealed that age (odds ratio, 1.009; 95% confidence interval, 1.002-1.015; p=0.010), heart failure (odds ratio, 1.765; 95% confidence interval, 1.448-2.151; p<0.001), smoking (odds ratio, 1.762; 95% confidence interval, 1.441-1.153; p<0.010), chronic kidney disease (odds ratio, 2.057; 95% confidence interval, 1.494-2.833; p<0.001), and deep vein thrombosis (odds ratio, 0.463; 95% confidence interval, 0.229-0.718; p=0.001) were independent predictors of combination therapy (r^2^=0.66). The mean time in therapeutic range of patients receiving combination therapy was significantly lower than in those on warfarin monotherapy (51.6±27.05 vs. 54.7±23.93; p=0.006). Overall, 19.4% (n=677) of patients had a bleeding event (major bleeding 13.0%, n=88) within a year. Percentages of patients with combination therapy were significantly higher in patients with major bleeding than in patients without major bleeding (29.5% vs. 19.7%; p=0.023).

**Conclusion:**

Our study demonstrated that 20.0% of patients taking warfarin use concomitant aspirin inappropriately in daily practice. Patients receiving aspirin with warfarin were demonstrated to have more comorbidities, lower time in therapeutic range levels, and higher bleeding rates.

Warfarin is used commonly in atrial fibrillation (AF), mechanical heart valve (MHV) replacement, and other diseases such as chronic pulmonary embolism, ischemic stroke, and deep vein thrombosis (DVT). Aspirin in addition to warfarin can be used to improve antithrombotic activity. Although the combination of aspirin and warfarin results in a significant increase (up to 1.5- to 2-fold) in the risk of bleeding, it does not add any benefit in stroke and cardiovascular events (1,2). Both the AF guidelines of the European Society of Cardiology (ESC) and American College of Cardiology/American Heart Association (ACC/AHA) do not recommend adding aspirin to warfarin treatment except in patients who are receiving warfarin for AF and have had an acute ischemic event or undergone coronary revascularization during the last year (3,4). However, for patients with MHV, there are different recommendations between the ACC/AHA and ESC guidelines regarding the use of aspirin in addition to warfarin. The ACC/AHA valvular heart disease guideline recommended that aspirin should be routinely added to warfarin therapy (class 1, level a). However, the ESC valvular heart disease guideline does not recommend adding aspirin to warfarin therapy routinely. It recommends that low-dose aspirin (75-100 mg/day) may be considered in the case of concomitant atherosclerotic disease (class 2b, level c) or should be considered after thromboembolism despite an adequate international normalized ratio (INR) (class 2a, level c) (5,6). The existence of such significant differences between the two guidelines can lead to physicians having different treatment recommendations for patients with MHV. Besides this difference, the inappropriate use of aspirin with warfarin is frequently among patients who take them concomitantly for any reason as directed by both community-based prescribers and randomized studies (7,8,9,10,11,12). Against this background, the objectives of the present study are to evaluate the prevalence of the inappropriate combination of aspirin and warfarin and to describe which patients are currently receiving combination therapy in daily practice. For this purpose, the data of the The awareness, efficacy, safety, and time in the therapeutic range of warfarin in the Turkish population (WARFARIN-TR) study were evaluated.

## MATERIALS AND METHODS

The main results of the WARFARIN-TR study have been published previously (13,14). In summary, The WARFARIN-TR study included 42 centers from 24 cities in Turkey from January 1, 2014, to December 31, 2014. The study protocol was approved by the local ethics committee. During routine clinical follow-up, the patients’ data were recorded. The INR values of patients were recorded from digital hospital records. All consecutive patients (n=4987) using warfarin regularly for any reason and attending routine INR monitoring were entered into the study. Patients who were not followed-up regularly, and under 18 years old were excluded from the study. For patients with warfarin due to non-valvular AF (NVAF) and other reasons, the target INR was 2.5 (range, 2.0-3.0). Also, the target INR value was 3.0 (2.5-3.5) for patients with MHV. The time in therapeutic range (TTR) was calculated according to F.R. Roosendaal’s algorithm with linear interpolation (15). The CHA_2_DS_2_-VASc [heart failure (HF), hypertension, age ≥75, diabetes, stroke, vascular disease, age 65-74 years, female sex] score of patients with NVAF was calculated at the time as the interview (3). Each risk factor except age ≥75 (2 points) and stroke (2 points) at this score is one point. Transfusion of at least 2 units of blood, reduction in the hemoglobin level of at least 2 g/L, or symptomatic bleeding in a critical area were determined as major bleeding. Other bleeding was considered minor bleeding. All consecutive patients who were using warfarin for any reason during the last year were included in the WARFARIN-TR study without considering whether there were any acute ischemic events or revascularization during the last year. Hence, it is not clear in the WARFARIN-TR study how many patients had acute ischemic events or revascularization during the last year. Therefore, for the present study, all patients who had a history of atherosclerotic disease (ischemic heart disease, peripheral artery disease) or cerebrovascular disease were excluded from the study (n=1498; 30.03% of all patients). The data of the remaining 3489 patients that did not have an indication for aspirin use under the direction of the ESC guidelines were analyzed. Although it is not proven by a study, the recommendations of the ESC guidelines are more applied in daily practice in Turkey than are the ACC/AHA guidelines. Because of this, in the present study, we have defined combination treatment as inappropriate for all patients who received aspirin and warfarin regardless of the warfarin use indication, under the direction of the ESC guidelines recommendation.

### Statistical analysis

Continuous variables were presented as the mean ± standard deviation, and categorical variables were expressed as number and percentage (%). Continuous variables were compared with the Student’s t-test or the Mann-Whitney U Test. A chi-square test or Fisher’s exact test was used to compare categorical variables. The homogeneity of variance was tested with Levene’s test. Independent predictors of combination therapy were determined by logistic regression analysis. Possible factors that were identified with univariate analyses were entered into the logistic regression analysis. The Statistical Package for the Social Sciences (SPSS for Windows, Version 20.0, SPSS Inc., Chicago, IL, USA) was used for the statistical analysis. A p value less than .05 was accepted as statistically significant.

## RESULTS

A total of 3489 patients (males 41.8%, mean age 59.2±13.8 years) were included. The prevalence of combination therapy was 20.0%. The primary indication for warfarin use for MHV, NVAF, and other reasons were 47%, 35.5%, and 17.5%, respectively. The prevalence of combination therapy in patients using warfarin due to MHV, NVAF, and other reasons was 20.5%, 18.7%, and 21.0%, respectively. The demographic characteristics of the warfarin monotherapy group and combination therapy group are summarized in Table 1. The combination therapy group was found to be older and had a higher prevalence of male gender and history of smoking, HF, and chronic kidney disease (CKD). There was no difference between the groups regarding the indication for warfarin use. The mean TTR (51.6±27.05 vs. 54.7±23.93; p=0.006) and number of INR examined during one year (9.65±3.08 vs. 10.5±3.39; p<0.001) were significantly lower in the combination therapy group when compared with the warfarin monotherapy group (Table 2). The mean CHA_2_DS_2_-VASc score was 2.8±1.29 in patients with NVAF, and there were no significant differences between groups. Although the CHA_2_DS_2_-VASc score was 0, 36 patients with NVAF had been prescribed warfarin, and 6 of these patients received combination therapy. Moreover, 26.5% (36/136) of patients with a risk score of 1, and 17.8% (117/996) with a risk score of ≥2 were receiving combination therapy. Multivariate logistic regression analysis revealed that age [odds ratio (OR), 1.009; 95% confidence interval (CI), 1.002-1.015; p=0.010], HF (OR, 1.765; 95% CI, 1.448-2.151; p<0.001), smoking (OR, 1.762; 95% CI, 1.441-1.153; p<0.010), CKD (OR, 2.057; 95% CI, 1.494-2.833; p<0.001), and DVT (OR, 0.463; 95% CI, 0.229-0.718; p=0.001) were independent predictors of combination therapy (r^2^=0.66) (Table 3). Overall, 667 (19.4%) patients had a bleeding event (13.0%, n=88, major bleeding). Demographic characteristics of patients with and without major bleeding are summarized in Table 4. There were no significant differences between patients with and without major bleeding except combination therapy and a history of CKD. Percentages of patients with combination therapy were significantly higher in patients with major bleeding than in patients without major bleeding (29.5% vs. 19.7%; p=0.023). Also, the mean TTR values of patients with major bleeding were significantly lower than the mean TTR values of patients without major bleeding (51.0±23.60 vs. 54.2±24.64; p=0.027).

## DISCUSSION

The present study has determined that one-fifth of patients who receive warfarin for any indication are receiving aspirin inappropriately in daily practice. In addition, patients with combination therapy were more likely to be older with a history of HF, CKD, and smoking. Furthermore, patients with combination therapy were more likely to have major bleeding compared with warfarin monotherapy. Both ESC and ACC/AHA AF guidelines do not recommend aspirin in addition to warfarin except in patients who have had an acute ischemic event or undergone coronary revascularization during the last year (3,4). Similar to AF patients, there is no recommendation to administer or prescribe aspirin in addition to warfarin to patients who are on warfarin for other reasons. However, although there is no recommendation, our results had shown that one-fifth of patients who use warfarin for AF or other reasons were receiving combination therapy inappropriately. Previous studies have shown that the combination of aspirin and warfarin did not lead to reduced stroke or vascular events, and resulted in an increased risk of bleeding in AF patients (16,17). However, randomized trials that evaluated new oral anticoagulants have demonstrated that 20%-36% of patients receiving warfarin for NVAF also received aspirin concomitantly (18,19,20). In these studies, a remarkable percentage of patients receive aspirin without having a history of atherosclerotic disease. Previous studies had shown that widespread use of combination therapy appears to be because of the presence of multiple comorbidities (7,18,19,20). Similar to these previous studies, our results showed that the presence of comorbidities such as HF, CKD, smoking, and older ages were strong independent predictors of combination therapy. Smoking, CKD, and older age are known to be risk factors for the development of cardiovascular disease. Similarly, HF has a high potential for the development of cardiovascular events. For this reason, when physicians prescribe combination therapy for a potentially complementary antiplatelet and anticoagulant action, they should consider the comorbidities or risk factors that may cause cardiovascular events. However, we were unable to determine whether patients received combination therapy as directed by a physician or their own initiative. As it is widely believed that aspirin is beneficial for reducing the risk of heart attack, and it is easy to get aspirin in Turkey without a prescription, many patients may have been self-administering aspirin as part of their therapy without the knowledge of their physician. On the other hand, when the CHA_2_DS_2_-VASc score was taken into account, our results showed that many patients with warfarin for NVAF receive warfarin or combination therapy even though they do not have an indication. This result shows that some physicians do not consider the CHA_2_DS_2_-VASc score that is recommended by the guidelines for NVAF patients when starting warfarin therapy. ESC Valvular Diseases guideline recommends adding low-dose aspirin to warfarin treatment in patients with simultaneous MHV and atherosclerotic heart disease (Class 2a) (21). The routine addition of aspirin to warfarin in patients with MHV, however, is not recommended. Despite the restricted indication of the ESC guideline, ACC/AHA valvular disease guideline recommends low-dose aspirin in all patients with MHV (Class 1) (21,22). In their study with 4557 patients, Johnson et al. (23) found that 47.8% of patients who were taking warfarin for AF, 27.4% for venous thromboembolism, 11% for MHV, and 6.6% for stroke or transient ischemic attack, 38.5% were receiving inappropriate combination therapy according to the American College of Chest Physician guideline.

In their study with 360 AF and MHV patients, Turan et al. (24) noted that 25.8% of the patients were receiving combination therapy. The same study also observed that 21.1% of AF patients and 32.7% of MHV patients were receiving combination therapy. In our study, similar to other indications, 20.5% of patients taking warfarin for MHV were using aspirin concomitantly. Different recommendations listed by the ESC and ACC/AHA guidelines for MHV patients regarding whether to use aspirin regularly or not might result in discrepancies among physicians who are following these guidelines. When evaluated from the perspective of the ESC guideline, there appears to be a high rate of inappropriate aspirin use among patients. When evaluated from the perspective of the ACC/AHA guideline, it appears that very few patients are receiving aspirin. However, as we mentioned above, the recommendations of the ESC guidelines are applied more in daily practice in Turkey when compared with those of the ACC/AHA guidelines. For this reason, our results have indicated that one-fifth of patients on warfarin for MHV receive aspirin inappropriately. As mentioned above, concomitant comorbidities are predictors of combination therapy use. Also, lower TTR levels might be a reason for combination therapy. Physicians might have preferred aspirin in patients with lower TTR to an agent with better antiplatelet and anticoagulant action. Similarly, the lower number of INR examined in one year demonstrated that these patients do not have regular follow-ups indirectly and physicians might have preferred combination therapy for these individuals. Bleeding is one of the most important events in patients treated with warfarin. Studies have shown that adding aspirin to warfarin did not reduce stroke, systemic embolism, and myocardial infarction, but rather, led to an increased risk of bleeding (25,26,27). Similar to previous studies, we demonstrated that the number of both major and minor bleeding events is significantly higher in patients with combination therapy. Also, combination therapy was a predictor of major bleeding. New-generation oral anticoagulants, which can be alternatives to the long-used warfarin, are currently being used. These drugs have significant advantages of not requiring close follow-up, not having food-drug interactions, and causing less bleeding with similar efficacy (9,10,18,28). New-generation oral anticoagulants with proven efficacy may be preferred instead of adding aspirin to warfarin to decrease the risk of thromboembolism in patients who fail to achieve effective TTR values or cannot attend regular follow-ups.

The most important limitation of this study is the lack of evaluation of events, such as stroke and myocardial infarction, in patients who experienced them during the course of the study.

Our study, which reflects daily practice, demonstrated that a high proportion of patients taking warfarin concomitantly use aspirin inappropriately. Patients receiving aspirin with warfarin were shown to have more comorbidities, lower TTR levels, and higher bleeding rates.

## Figures and Tables

**Table 1:**
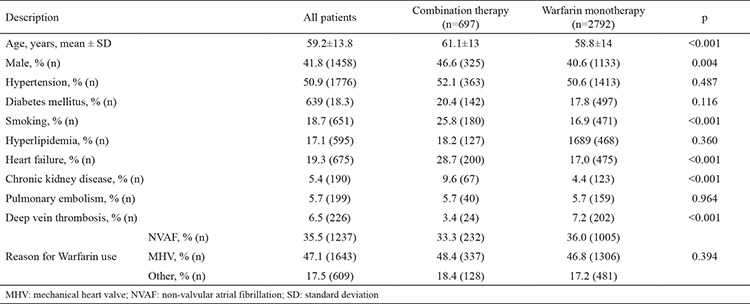
Comparison of baseline characteristics of patients with combination therapy and warfarin monotherapy

**Table 2:**
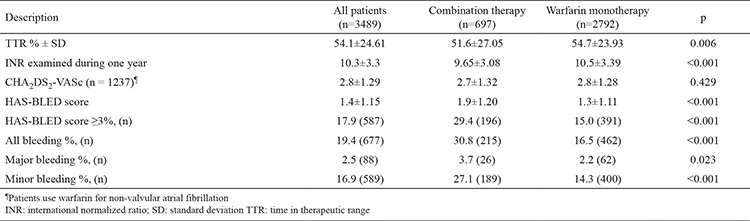
Comparison of laboratory results, risk scores and bleeding complications of the patients with combination therapy and warfarin monotherapy

**Table 3:**
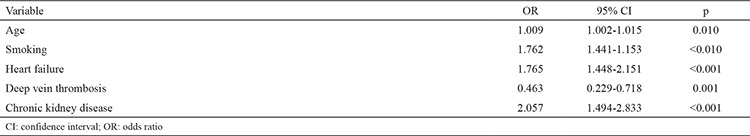
Predictors of combination therapy in multivariate logistic regression analysis

**Table 4:**
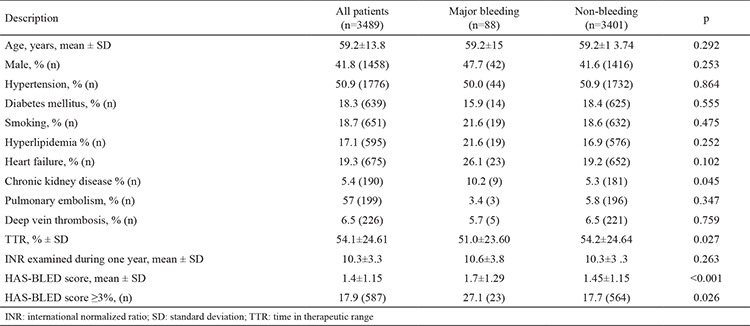
Comparison characteristics and laboratory parameters of patients with and without major bleeding
